# In Limbo: Seven Families’ Experiences of Encounter with Cancer Care in Norway

**DOI:** 10.5334/ijic.5700

**Published:** 2021-11-26

**Authors:** Monica Solberg, Geir Vegard Berg, Hege Kristin Andreassen

**Affiliations:** 1 Norwegian University of Science and Technology and Innlandet Hospital Trust, NO; 2 Norwegian University of Science and Technology and The Artic University of Norway, NO

**Keywords:** cancer pathways, family, qualitative research, integrated care, patient-centred care

## Abstract

**Introduction:**

Like many other countries, Norway has seen a shift from inpatient to outpatient cancer care, with pathways aimed at improving the integration and coordination of health services. This study explores the perspectives of seven patients and their family members in light of this change. We focus on one particular phase of the pathway: the first encounter. Our interviews were set in the period from referral until the start of treatment.

**Methods:**

Nineteen individual in-depth interviews were conducted in seven families. Seven patients with cancer and 12 family members were interviewed.

**Results:**

Three categories of experiences stood out in the empirical material: ‘Being in between different health professionals’, ‘Overwhelmed by written and oral informationʼ and ‘Lack of involvement’.

**Conclusion:**

This study provides insight into families’ experiences with cancer care from referral until the start of treatment. Our findings indicate that families often experience cancer care as fragmented and confusing. Although evaluations have shown that the introduction of cancer pathways seems to have a positive effect on waiting times and standardization of examinations across hospitals and regions, there is still potential for improvement in coordination between services, family involvement, and emotional and practical support. We argue that our findings highlight the tension between two ideals of professional care: standardization and patient-centredness. The study illustrates shortcomings in translating the ideal of patient-centredness into professional practice.

## Introduction

In 2040 the global cancer burden is expected to rise 47% from 2020 [1]. In 2019, more than 283,000 people in Norway have or have had cancer [2]. Norway, with a population of five million, has seen a shift to outpatient services in cancer care. Hospitalizations are fewer and shorter than before. Those affected by cancer spend more time at home, where home care nurses (HCNs) and general practitioners (GPs) are responsible for care [3]. When a patient is diagnosed with cancer, the person who is diagnosed is not the only one affected – the lives of family members are also changed [4].

National and international studies indicate that overall cancer care delivery is inadequate [5,6,7,8]. This criticism addresses aspects of coordination and collaboration between and within health service levels [7,8,9], and the involvement of the patient and family [10]. Therefore, 28 cancer care pathways were introduced in Norway in 2015, aimed at better integration and coordination of health care services. A cancer pathway includes assessment, initial treatment and follow-up, and treatment of any relapse. This study follows the informants through the assessment period from receipt of the referral by the hospital until a decision on treatment has been made. Assessment comprises three phases, each with a specific timescale [11].

Cancer pathways are standardized patient processes that describe the organization of the assessment and treatment, communication/dialogue with the patient and the family, as well as the allocation of responsibilities and specific process times. The intention is to reduce processing times, speed up diagnosis and onset of treatment, and ensure that all patients are treated according to national clinical guidelines. Patients and their families should experience well-organized cancer care, holistic treatment, and a predictable progression [11].

In recent decades, several national reforms and various strategies have been introduced to improve the healthcare system [3,12] and cancer care in Norway [5,11]. These emphasize ‘coordination between and within the levels in health care’ and ‘patient-centred treatment’ as important elements in achieving integrated patient care. In the literature, there is a discussion of whether it is possible to create systems that reconcile coordination of care with a patient-centred approach /successfully combine coordination of care with a patient-centred approach [13]. Patient-centred treatment strives for adaptation to each patient’s values and preferences. In contrast, coordination often entails standardization in the form of automated procedures and streamlining of health care across cases [13,14].

Our research highlights end-user experiences of cancer care organization and coordination in Norway, i.e., the integration of care as experienced from a patient and family perspective. The concept of integrated care, as the opposite of fragmented systems, is not commonly used in cancer care. Nevertheless, there are more than 175 overlapping definitions and concepts of ‘integrated care’ within organizational and system theory [15]. Most definitions refer to a care organization that follows a logic of integration to improve the outcome for the target group by improving coordination both within and between the various health systems [16]. In the cancer research literature, integration is mainly used to describe various initiatives to improve aspects of cancer care [16]. Although cancer patients receive treatment and/or follow-up at all levels of health care, less attention is given to initiatives within integration that enhance coordination across service providers or levels in healthcare systems and to aspects of integration from the family and patient-centredness perspective [17,18]. This gap in research needs attention because the hospital setting represents only a very brief part of the experience, and follow-ups are increasingly shifted to outpatient care [19,20].

### Aim

The aim of the study was to describe and explore how the cancer patient and their family experienced being in cancer care, through the assessment period from receipt of the referral by the hospital until a decision on treatment has been made.

## Method

### Design

This study used a qualitative design to explore families’ experiences with the health care system and everyday life before the start of treatment. Qualitative research methods are well suited to exploring people’s experiences, thoughts, expectations, and attitudes [21].

### Context of the study

**Figure 1:**
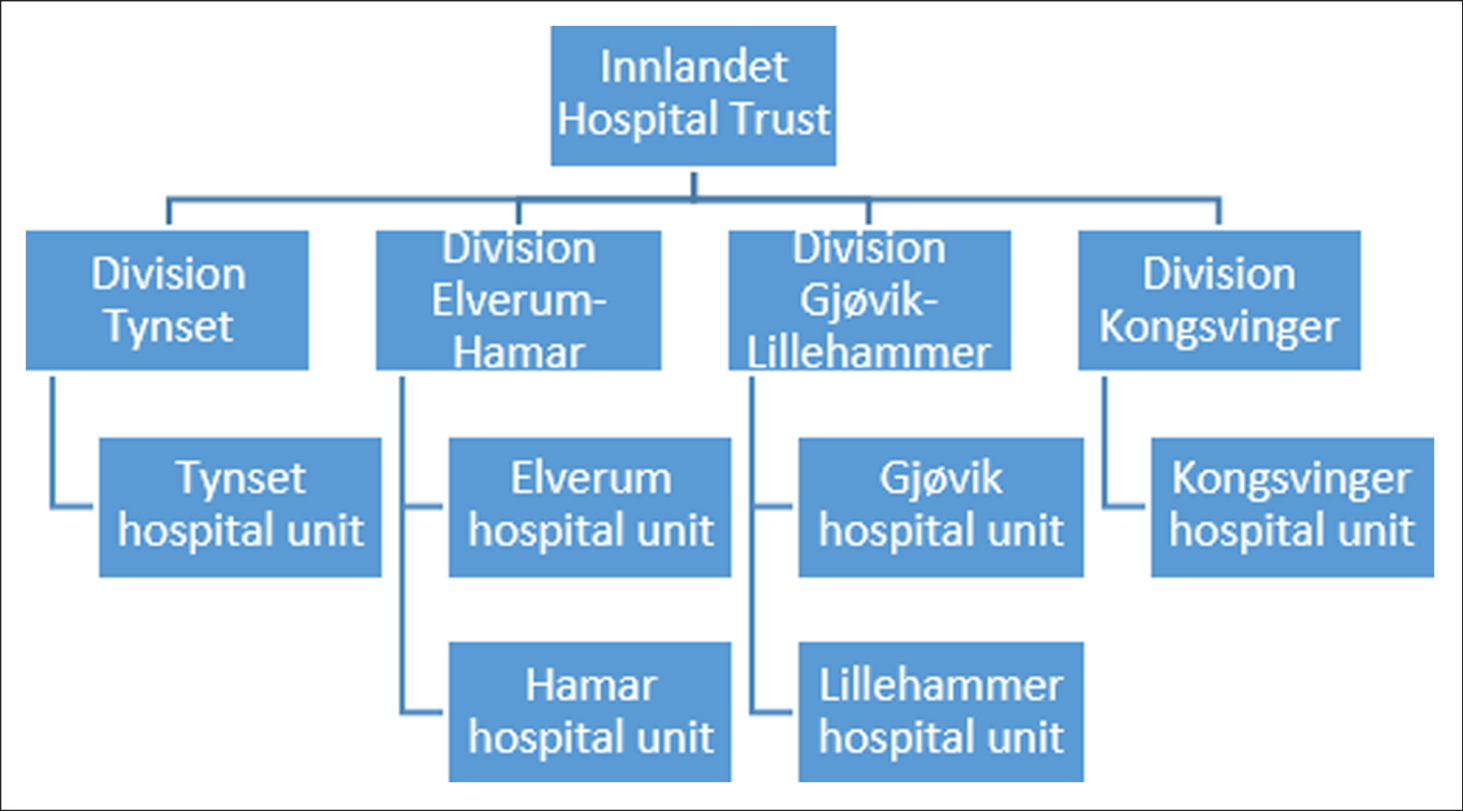
Organisation of Innlandet Hospital Trust.

Norway has a semi-decentralized health system with four regional health authorities (RHAs) with responsibility for specialist care and 422 municipalities responsible for primary care and social services [22]. This study was conducted in South-Eastern Norway RHA, which consist of ten local- and one regional Hospital Trust. The somatic care in the local Hospital Trust the study was conducted was organized into four division with six hospital units until January 2019 (see ***Figure 1***), with a catchment area of approximately 300 square kilometres.

All hospital units offer treatment for various cancers, but only two units perform nuclear medicine and only one performs radiation therapy. One unit introduced a new concept called ‘the same day’ principle during the study’s recruitment phase. The principle is that on the same day patients receive their diagnosis, they meet all the healthcare professionals who will be involved during the surgery. In our case, this appointment was with the surgeon and a nurse who also conducted the initial conversation, the plastic surgeon, the anaesthesiologist, and the laboratory personnel.

### Recruitment and sample

All heads of departments at the medical outpatient clinics were contacted to obtain approval for patient recruitment in their clinic. Both oral and written information about the project was provided to all hospital units. In addition, we met all the pathway coordinators to inform them about the project and get assistance for recruitment. Only two of the hospital units responded positively. The surgeon who informed the patient of the diagnosis also asked if they wished to participate in the study. The first author spoke to patients immediately after the consultation and gave them oral information about the study as well as written information that they could discuss with their family. After a few days, the informant or the researcher confirmed whether they wanted to participate in the study or not. Seven families agreed, and six families declined. Three of the families who did not participate in the study justified this because they did not have the energy and capacity to participate. The other three gave no reason.

Recruitment was by strategic selection of informants who possessed certain characteristics and qualities in line with the study’s objective [23]. The inclusion criteria for patients were that they had a confirmed cancer diagnosis, spoke and understood Norwegian, were competent to give informed consent and were over 18 years old. The patients defined who their family was. The inclusion criteria for family members (FMs) were that they spoke and understood Norwegian, were competent to give informed consent and over 16 years old. ***Table 1*** shows the characteristics of the participants.

**Table 1:** Characteristics of patients and family member.


FAMILY	PATIENT AGE	SEX OF PATIENT	FM’S RELATIONSHIP TO THE PATIENT	HEALTH CARE LEVELS AND UNITS	HOW THE CANCER WAS DISCOVERED	TIME FROM DIAGNOSIS TO START OF TREATMENT

Family 1	>60	Female	Two sons	GP, two local hospital units and five departments	Suspicion of another disease	Six weeks

Family 2	51–60	Female	Partner and daughter	Two local hospital units and six departments	Via BreastScreen Norway*	Ten days

Family 3	41–50	Female	Husband	GP, two local hospital units and six department	Felt a lump in the breast	Two weeks

Family 4	51–60	Female	Aunt and friend	GP, two local hospital units and six department	Felt a lump in the breast	Two weeks

Family 5	>60	Female	Son and daughter	GP, two local hospital units and seven departments	Was hospitalized with another disease	One week

Family 6	51–60	Female	Husband	GP, two local hospital units	Felt a lump in the breast	One week

Family 7	51–60	Female	Partner and daughter	GP, two local hospital units and seven departments**	Via BreastScreen Norway*	Three days


* BreastScreen Norway offers screening mammography to all women between the ages of 50 and 69 every other year, and the participation is voluntary. ** Department at the local hospital units.

### Data collection

Individual in-depth interviews were used for data collection. The interview was semi structured, and based on a thematic interview guide consisting of one opening question and a series of follow up questions sorted into four topics. The opening question was: ‘Can you tell me your story, from the time you suspected something was wrong until now?’ The four topics were everyday life; information from the hospital, family, and environment; quality of life; follow-up during the waiting phase. The interview guide was tested on two user representatives and then developed with input from them. The material was digitally recorded by the first author and transcribed verbatim by the first author and a professional transcriber. The quotes in this article have been translated from Norwegian to English by a professional translator.

The interviews were conducted by the first author from June 2018 until February 2019. The semi structured method allowed for informants to talk freely. In most of the interviews, the informants covered the four topics of the interview guide themselves, without input from the interviewer. The participants chose when and where the interviews would be conducted. Thirteen took place in their home, five took place in an office at the hospital and one via Skype. Each interview lasted from 20 to 60 minutes (average 44 minutes).

### Ethics

The informants received oral and written information about the study. Written informed consent was obtained and the informants were told that they could withdraw from the study at any time. Data in the study were anonymized, kept confidential and stored on a secure server. The Regional Committee for Medical and Health Research concluded that the study was not regulated by the Health Research Act (2016/1486/REK sør-øst C). The study was approved by the Norwegian Centre for Research Data (NSD) (project number 51466) and was conducted in accordance with research ethics guidelines [24,25].

### Analysis

The analysis of the transcribed material is inspired by systematic text condensation [26]. The analysis proceeded in the following manner; First, each of us read and did a preliminary coding of the interviews, individually. We then met for a workshop to do a comparison and discuss how to group our codes into categories and themes. After this first workshop we agreed to pursue the overall main theme “Logistic and organisation”, which all of us had noted as a core theme in the interviews. We went back to the interviews for a more thorough reading, and now coded and re-coded all quotes that were related to this overall theme. Through a series of meetings between the researchers involved, these codes were then synthesized into three categories: being in between different health professionals; overwhelmed by written and oral information; and lack of involvement. See ***Table 2*** for an example of the analysis.

**Table 2:** The analysis process.


STEP 1: TOTAL IMPRESSION – FROM CHAOS, VIA PRELIMINARY CODING, TO OVERALL THEME	STEP 2: BACK TO THE INTERVIEWS: CODING AND RE-CODING	STEP 3: FROM CODES TO CATEGORIES

Logistics and organization	Do not have a clear overview Must relate to several hospitals Do not trust the local hospital Not sure if the GP will be updated	Being in between different health professionals


## Findings

Our findings illuminate family experiences of the first phase of cancer care in the Norwegian cancer pathway. Through our analysis we identified concerns about logistics and organisation as an overall theme, characterizing this phase for all families we talked to. The different aspects of the experiences shared with us on this theme can be grouped into three categories: experiences of ‘Being in between different health professionals’; experiences of being ʻoverwhelmed by written and oral informationʼ; and experiences of ʻlack of involvementʼ.

### Being in between different health professionals

All families explained that they lacked an overview of what was happening, as they had to deal with several professionals from different units. All the patients had to go to two local hospital departments before starting treatment. The fragmentation of cancer care among several hospital departments was perceived as confusing and worrying because they had to relate to several different therapists. Furthermore, they were uncertain whether their GP had up-to-date knowledge or not. One patient elaborated:

(…) when I think about having to visit first one local hospital to get something done, and then another local hospital to get something else done, and after that, I have to go home and keep going, and my GP, who’s supposed to help me from then on, doesn’t know what’s happened (P5).

The patients were frustrated because their GP had no information about their hospital patient record. This led to patients asking the hospital for transcripts from the appointments and examination that they could give to their GP.

Although the patients reports that the time from the consultation with their GP until they were examined at the hospital had gone very fast. The time from diagnosis to surgery was also experienced as short. The patients perceived this as positive, as they did not have much time to worry. Furthermore, they said that waiting was both stressful and tiring. One patient said that she became a little worried that things were going so fast:

I was almost a little scared right away when she said I was already set up for surgery, I thought my god, this is really serious, since they have to take it so fast (P7).

The families had previous experience of both local and regional hospitals. They expressed concern about whether the local hospitals had the same professionalism as the regional hospitals and the necessary capacity and competence to deal with life-threatening illnesses. Furthermore, some families mentioned negative experiences with the local hospitals, which led to mistrust, and stated that they were less professional than the regional hospitals. One FM expressed this as follows:

So of course, I’ve experienced all the professionalism they have at the regional hospital … and the nurses there always made the time … When the nurses came in, we could ask them questions, and they stopped and took the time to explain (FM6).

The patients who were involved in the new ‘same day’ concept found it chaotic and confusing. First, they had been told that they had cancer, and then they had to talk to several doctors. This led to a lot of waiting and information overload in one day. One patient described this as follows:

We had barely sat down when the doctor said it was as we thought, that there were cellular changes, so it was breast cancer. Yes, OK. That’s clarified. Check. What do you want to do now? Eh, what? Is it up to me to decide? And then after ten minutes, the plastic surgeon came in … and then it was the anaesthesiologist … and finally a blood sample and then chucked out … and we’d only been there for two hours … that’s kind of how I felt (P7).

This patient had her husband with her, which she greatly appreciated because they remained in the hospital for over two hours. Another patient did not bring anyone to the consultation because she wanted to spare her loved ones in case she received bad news, but at the next appointment at the hospital, she brought her partner.

### Overwhelmed by written and oral information

The patients perceived the information they received before they received the diagnosis as vague. All of them said that they wished that the hospital had recommended them to bring someone to the consultation, and that the information could have been a little more direct and straightforward. Some of the patients did not bring anyone with them to the hospital appointment because they did not want to bother anyone and realized afterwards that this had been a mistake. When a patient receives all the information on their own, it is difficult to remember it all, and then even harder to inform FMs. The patients also said they were unsure if they had understood the information correctly. One FM reacted as follows:

I think that’s totally screwed up. That shows they don’t understand what happens to somebody who’s just been told that it’s malignant, so it’s cancer. Just hearing that word. It’s something that scares most of us, isn’t it? I don’t get it, that they can act in that way. What do they think people are made of? (FM6)

Bringing someone to the consultation was reported as positive. The patients said when they were two, they complemented each other with respect to the information they received, and after the consultation they could discuss the information and fill each other in. Some of them had no plans to bring anyone, but they were glad they had, since they felt they would never have been able to absorb all the information on their own.

The patients said that they wished they had received a written account of the conversation so they could read through it afterwards with their family. Everyone had received an information letter that described their type of cancer in general and listed a contact person at the local hospital with their telephone number. Furthermore, the patients said that they had not received any information about available resources in their municipality. One of the patients had tried to call the number provided but did not make contact for several days. She commented:

I actually called for three days before I finally got hold of her, and I think that was actually pretty strange because it was her direct phone number … they should have an answering machine or a mobile phone so they can see that someone has called and then call back. I started to call her on Wednesday and finally got hold of her on Friday. I think that’s reprehensible (P4).

Both the patients and the FMs said that they chose to google information rather than contacting the hospital, although they felt this was not the best way to get concrete information. They did so because they did not want to bother the healthcare professionals with minor questions. The patients said the hospital could have called them a few days after the consultation or given them an SMS number or an email address where they could send questions. They wanted answers to practical questions such as: ‘Should I come to the hospital the day before or the same day as the operation?’ ‘Is it one night or more?’ ‘Is there accommodation for any family member?’ ‘Can I drive myself?’

### Lack of involvement

The patients said that they did not feel involved in the decision about the treatment. The information given during the consultation was described as concrete and understandable, but afterwards they had many questions. The doctor did not discuss different treatments with them but only stated which treatment decision had been made. Furthermore, they were not informed about the advantages and disadvantages of different types of treatment. Viewing the x-rays was mentioned as an example of getting involved. A patient with breast cancer explained the benefit of seeing the images:

(…) If the surgeon had said “Look, here’s an image of your breast, and here’s the lump, and this is what we’re thinking. Then I would have understood the whole picture better (P3).

FMs described being ignored in meetings with health professionals who addressed information to the patient and not to them. They wanted to be involved and informed in order to help the patient physically, practically and psychologically, as well as to have a contact person to call if they had any questions. One patient explained how important it was that the family was involved and informed:

I think it’s vital that the family is involved. I think you recover faster. Things go better then, and you take more care of each other (P5).

The FMs said that they had no expectations of being heard and involved by the health personnel – they felt they were only passive observers.

## Discussion

The aim of this study was to explore families’ experiences of the interaction with professional cancer care in Norway during the first phase of the cancer pathway: from referral until treatment starts. The context for our study are the recent changes in organizing cancer care with well-defined pathways, with the intention of better integrating and coordinating health care services in cancer care. In the interviews, the cancer patients and their families revealed what was important to them and why. Our analysis identified three main categories of experiences: being in between health professionals; overwhelmed by written and oral information; and lack of involvement.

To support our empirical exploration of patient experiences in the encounter with cancer care, we found it useful to draw on two theoretical discussions in the health sciences: how to understand integrated care and the discussion on patient-centredness in health care.

The introduction of pathways involves standardization of cancer care. Standardization has been described as contributing to objectifying and thereby alienating the patient, instead of making them an active participant with individual subjective contributions [18]. Patient-centredness on the other hand, has been described as care that is responsive to patient preferences, needs and values, which includes integration and coordination. Emotional and physical support is provided and allows for the involvement of friends and family. Furthermore, information and knowledge are provided to enable the patient to make decisions about their own situation and care [19].

Integrated care, meaning coordination and collaboration that includes involvement and support for FMs, are mentioned in national reforms, legislation and strategies, but what does the encounter with cancer care look like in practice, from the patient’s perspective? Do patients and their families experience integrated care and involvement? Moreover, do they experience the services as coordinated, open to their values and preferences, providing psychological and emotional support, and safeguarding both patients and the family?

### Integrated cancer care

In Norway, cancer pathways constitute an important part of integrated services in current cancer care. The aim of the cancer pathways, introduced in 2015, was for patients and their family to experience ‘well-organized cancer care, holistic treatment and predictable progression’ [11].

A survey conducted before and after the introduction of the Cancer Pathways Programme revealed little collaboration and coordination between the various actors in the health system [27]. Patients’ comments to us on how they had to deal with numerous health professionals from several departments, local hospitals and levels of the health care system confirm previous findings from Norway [27]. When they met with new health care staff, the new professionals they met were often not ‘up to date’ on their medical history. Of particular interest to the discussion in our study is the survey result that collaboration between GPs and the hospital was rated as especially poor [27]. Our study supports this finding. We were told stories of how patients had to update their GP about the results of examinations, their appointment at the local hospital, and specialists’ plans for follow-up. In fact, patients themselves form an important part of the information flow between institutions and units. Still, most of the patients and FMs were positive about the timeframe between diagnosis and the beginning of treatment. Others felt concerned about the speed of the process, which led them to think their diagnosis was more serious than it was. Similar findings were found in a Norwegian study on how patients experienced waiting times within standardized cancer patient pathways [28].

Other studies in which both patients and health care professionals perceived a lack of cooperation between hospital staff and GPs present similar findings [8,28]. According to Singer’s framework for studying integrated care, this dimension should be analysed to assess whether the service provided by the patient’s care team is consistent with other services the patient receives, and that there is a transfer of information [18]. From the literature, we know that fragmented care can lead to delays in treatment and ambiguity in the relationship of responsibility [6], as well as distrust and insecurity [29].

None of our interviewees had received information about available resources in their municipality. This was despite their respective municipalities offering services from a cancer nurse and local cancer association. Furthermore, informants agreed that they would have appreciated a contact point during the waiting time between diagnosis and the start of treatment, such as an SMS number or email address to which they could send questions. Some also mentioned that they would have appreciated a phone call from health personnel a few days after the consultation. Similar results were found in a US study, where patients and caregivers expressed a need for alternative communication such as email, telephone and text messaging in between appointments with various contact points in the health care system [7].

### Patient-centred dialogue or standardized information

In our study, we found that the families in general were informed about the diagnosis and treatment protocol. However, they were not involved in the decision making about the treatment protocol. They received copious information, but it was standardized and not adapted to the individual patient and family. In other words, they were not involved in dialogue, but informed in one-way communication. Patient–clinician communication increases satisfaction and is associated with patients feeling that they are informed and involved [30].

The informants in this study experienced that the doctors had already decided on treatment before the patient came to the first appointment. Patient care was not ‘tailored to the patient’s need and preferences’, in terms of the extent to which providers ‘consider the needs, preferences, values, and capabilities of the patient, family members, and other caregivers’ [13]. When patients are not allowed to participate in decisions about their own treatment, this may contribute to a feeling of being less involved [30]. Furthermore, they wanted more information about their condition, different treatments and their advantages and disadvantages.

The patients we interviewed also underlined that they wanted health professionals to use several forms of communication. At present, most of the information is given orally, during patient–provider appointments. Our informants explicitly stated that they would prefer to have information both orally and in writing. This would ease the communication with their FMs after the consultation and allow them to double check whether they had got it right the first time.

Similar findings emerged in a study of patients with stroke [31], where the patient experienced lack of involvement such as being informed about procedures, getting feedback on test results and being involved in decision making. Lack of knowledge can hinder patient participation and affect the balance of power between patients and health care professionals [32]. Patients involved in shared decisions report higher quality of communication and of the treatment they receive [33].

Another recurring theme in our material was that the patients who came alone to the consultation at the hospital failed to take in all the information and experienced information overload. When questioned directly, all informants answered that they wished they had been explicitly encouraged to bring someone. This finding is in line with those of other studies that have concluded that it is an advantage that the patient and their family get information together, as people in crisis perceive only parts of the conversation [34]. Other benefits mentioned were that FMs could ask questions, remember details of the conversation, receive important information, take notes, and report symptoms that the patient might have forgotten [34,35]. Well-informed patients are more likely to take part in their own care and treatment, which increases their participation [36,37,38]. In our study we found that patients who did bring someone to the consultation found that this helped them to remember and understand what was said, and to reflect on and sum up the content of the information perceived. It turned out that patients and FMs often perceived oral information differently and remembered different aspects of it.

In our study, the families reported that FMs were neglected/overlooked and not involved by health professionals, unless they asserted themselves. Both the written and oral information provided to the patients in our sample was directed to the patient, and not FMs. FMs often used the internet to get answers to their questions. Previous research has shown that FMs are less likely to feel overwhelmed and more likely to be more effective if they are involved in cancer care [39].

We found that the crucial elements for patients and their FMs in the first phase were good and consistent services from all professionals and institutions involved – including primary care services – and explicit involvement in decision-making. These findings are important as they can tell us how to ensure good cancer care that patients and FMs perceive as trustworthy. When encountering cancer care, patients experience system-centredness – as opposed to the well-publicized professional and policy goal of patient-centredness. We observe a tension between the standardization and efficiency focus in cancer pathway thinking and the aspiration to provide patient-centred care.

### Limitations and strengths

Interviewing people in a difficult situation raises several ethical questions. The first author had experience as a psychiatric nurse, so she had to be careful that the roles did not change from that of interviewer to that of therapist. Interviewers also had to be aware of their own experiences with the phenomenon and not ask leading questions. Our findings are based on a limited sample of those receiving cancer care in Norway. Our sample was limited to female cancer patients, with only two types of cancer. However, the sample size of this qualitative study was large enough to reach saturation [40,41]. To strengthen the trustworthiness of the study, two user representatives contributed throughout the process of designing project descriptions, interview guides and analysis.

## Conclusion

This study provides insight into family experiences of the encounter with cancer care during the very first phase of the cancer pathway – from referral until treatment starts. Our findings indicate that families often do not experience integrated care in this situation. Collaboration between and within the hospital and the GP seems to be important for the families in this first phase. Although evaluations have shown that the introduction of cancer pathways seems to have a positive effect on the time aspect and standardization of examinations across hospitals and regions, there is still potential for improvement in family involvement, coordination between services and emotional and practical support.

The themes that emerge in this study describe the need for better collaboration and coordination at a system level, and more involvement of patients and their FMs at an individual level. Thus, adjustments of Norwegian cancer care are still needed at both a system and an institutional level. Still, we believe that with small adjustments such as adding printed and digital information to the oral consultation, adding procedures for family involvement, and improving the routines and systems for information sharing between GPs and hospitals, some of these challenges could be minimized.
